# Ultra-Structural Imaging Provides 3D Organization of 46 Chromosomes of a Human Lymphocyte Prophase Nucleus

**DOI:** 10.3390/ijms22115987

**Published:** 2021-06-01

**Authors:** Atiqa Sajid, El-Nasir Lalani, Bo Chen, Teruo Hashimoto, Darren K. Griffin, Archana Bhartiya, George Thompson, Ian K. Robinson, Mohammed Yusuf

**Affiliations:** 1 Centre for Regenerative Medicine and Stem Cell Research, Aga Khan University, Karachi 74800, Pakistan; atiqa.sajid@aku.edu (A.S.); elnasir.lalani@aku.edu (E.-N.L.); 2 London Centre for Nanotechnology, University College London, London WC1H 0AH, UK; bo.chen@tongji.edu.cn (B.C.); a.bhartiya@ucl.ac.uk (A.B.); i.robinson@ucl.ac.uk (I.K.R.); 3 School of Materials Science and Engineering, Tongji University, Shanghai 201804, China; 4 Key Laboratory of Performance Evolution and Control for Engineering Structures of the Ministry of Education, Tongji University, Shanghai 200092, China; 5 Department of Materials, University of Manchester, Oxford Road, Manchester M13 9PL, UK; T.Hashimoto@Manchester.ac.uk (T.H.); george.thompson@manchester.ac.uk (G.T.); 6 School of Biosciences, University of Kent, Canterbury CT2 7NJ, UK; D.K.Griffin@kent.ac.uk; 7 Brookhaven National Laboratory, Upton, NY 11973, USA

**Keywords:** cytogenetics, chromosome, chromosome territory, microscopy, three dimensions, serial block-face electron microscopy, prophase

## Abstract

Three dimensional (3D) ultra-structural imaging is an important tool for unraveling the organizational structure of individual chromosomes at various stages of the cell cycle. Performing hitherto uninvestigated ultra-structural analysis of the human genome at prophase, we used serial block-face scanning electron microscopy (SBFSEM) to understand chromosomal architectural organization within 3D nuclear space. Acquired images allowed us to segment, reconstruct, and extract quantitative 3D structural information about the prophase nucleus and the preserved, intact individual chromosomes within it. Our data demonstrate that each chromosome can be identified with its homolog and classified into respective cytogenetic groups. Thereby, we present the first 3D karyotype built from the compact axial structure seen on the core of all prophase chromosomes. The chromosomes display parallel-aligned sister chromatids with familiar chromosome morphologies with no crossovers. Furthermore, the spatial positions of all 46 chromosomes revealed a pattern showing a gene density-based correlation and a neighborhood map of individual chromosomes based on their relative spatial positioning. A comprehensive picture of 3D chromosomal organization at the nanometer level in a single human lymphocyte cell is presented.

## 1. Introduction

Ultra-structural studies of the human genome have been commonplace since the Sutton–Boveri theory [1] and aimed at understanding the role of chromosomal structure and the spatio-temporal organization responsible for transmitting genetic information. The human diploid genome is comprised of approximately 6.4 billion base pairs (bp) of DNA packaged into 23 structural chromosomal pairs [2]. Each pair differs in size, with chromosome 1, containing 248.9 mega base pairs (Mbp), being the largest and chromosome 21, containing 46.7 Mbp, being the smallest [3]. As a cell progresses through the cell cycle, chromatin undergoes conformational changes from a decondensed state at interphase to a much more condensed state at metaphase [4,5].

The prophase stage of the cell cycle marks the beginning of mitosis. It involves remodeling, resulting in a gradual condensation of interphase chromatin into discrete and recognizable entities [6,7]. This is followed by aligning at the metaphase plate for correct segregation into a new daughter cell [8]. Although prophase chromosomes are observed before nuclear envelope breakdown, the condensation process involves a series of transitional events from early to late prophase into early prometaphase [7,9]. This high-order chromatin folding occurs with the assistance of several chromosomal structural and scaffold proteins [10,11,12,13] forming chromatin loops that tether the axial structure of the chromosome [14,15]. Chromatin in the cell’s nuclei during interphase has been shown to occupy non-random discrete chromosomal territories (CT) [16,17,18]. These CTs are further organized into different domains such as compartments (transcriptionally active A and transcriptionally inactive B) [19], topologically associating domains (TADS), and loops [20,21,22,23]. Cell cycle stage-specific differences in chromosome positioning have been observed in different cell types [23]. Chromosomal positioning has also been shown to influence the stability of the genome [24] and is implicated in different diseases including cancer [25], chromosomal translocations [26,27], gene expression patterns [28,29], differentiation [30,31], and development [32]. In late prometaphase, these territories have been shown to organize randomly [33] or non-randomly as parallel rosettes [34,35]. To date, our understanding of the structure and the 3D spatial organization of chromosomes at the prophase stage of the cell cycle remains sparse, as imaging is technically difficult without compromising the 3D structural integrity of the cell.

A limited number of studies have examined all 46 human chromosomes simultaneously using fluorescence in situ hybridization (FISH) [36,37]. More recently, chromosome conformation capture (3C) techniques mainly Hi-C (all with all contacts) has allowed mapping of genome-wide inter/intra chromatin interactions [38]. Three-dimensional (3D) advanced microscopy using serial block-face scanning electron microscopy (SBFSEM) has also been utilized to determine the structure and position of prophase chromosomes [39,40]. To date, however, only a small number of chromosomes in the prophase karyotype have been investigated and validated using SBFSEM [39,40].

To address this deficit, we present proof of principle for the ultra-structural (SBFSEM) imaging of a single cell at prophase, identifying all 46 chromosomes in human lymphocyte. Our data demonstrate the segmentation and identification of all chromosomes displaying a compact axial structure within the chromosome core. This approach allowed us to build the first 3D human prophase karyotype. We further demonstrate the spatial positioning of the chromosomes within the nucleus and their relationship to one another.

## 2. Results

### 2.1. Segmentation and Reconstruction of Chromosomes

**Figure 1:**
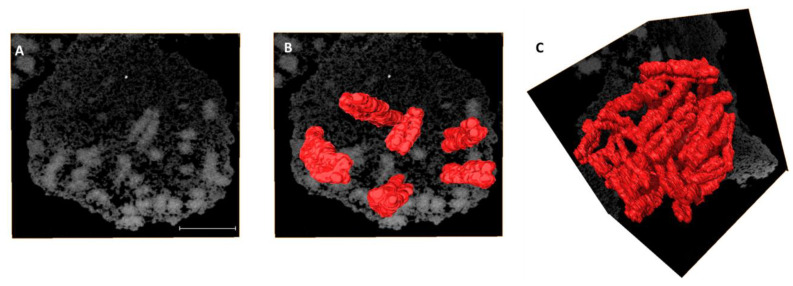
Volume reconstruction of individual human chromosomes from SBFSEM data. (**A**) A slice from an acquired SBFSEM stack of 345 backscattered electron (BSE) micrographs of a single prophase nucleus. Volume reconstruction of (**B**) 6 and (**C**) 46 chromosomes after segmentation. The pixel size of BSE micrographs is 11 nm × 11 nm and the sectioning thickness is 25 nm. Scale bar—2 µm.

Alignment of all SBFSEM slices allowed visualization of the single lymphocyte prophase nucleus. Two-dimensional (2D) sections displayed consistent contrast throughout the 3D stack, whereby rod-like structures representing chromosomes were seen (Figure 1A). As each chromosome showed structural variation in its 2D slices (Supplementary Figure S1A–C), manual segmentation was performed to allow 3D volume reconstruction of single chromosomes (Figure 1B, Supplementary Figure S1D), resulting in identification of all 46 chromosomes (Figure 1C). The lymphoblastoid cell line (GM18507) has 46 chromosomes [41,42] verified by mFISH karyotyping on metaphase chromosomes prepared from the 2D cultures (Supplementary Figure S2A–E).

### 2.2. Identification of All Individual 46 Chromosomes

**Figure 2:**
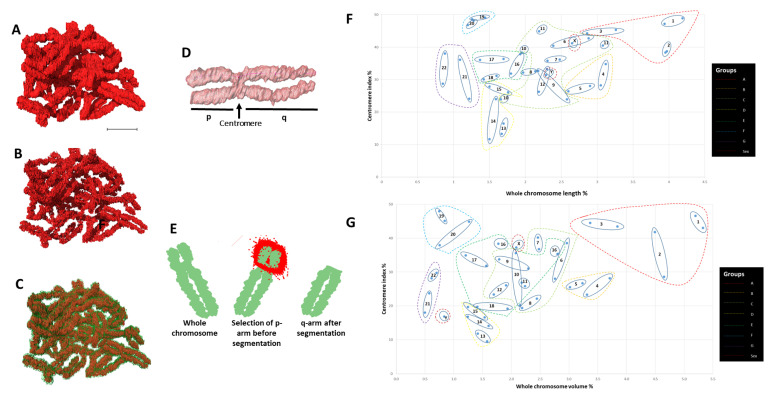
Identification of chromosomes. (**A**) 46 chromosomes after medium-segmentation. (**B**) 46 chromosomes after narrow-segmentation. (**C**) 46 chromosomes after medium (outer green color) and narrow (inner red color) segmentation from (**A**) and (**B**) respectively. (**D**) Length measurement of p and q arms of single chromosome. (**E**) Volume measurement of p and q arms of single chromosome. (**F**) Scatter diagram of eight groups (A to G and sex) labelled according to their assigned chromosome number. (**G**) Scatter diagram of centromere index against volume for all 46 individual chromosomes that are clustered after narrow-segmentation. Scale bar—2 µm.

Individual 3D reconstructed chromosomes (referred to as wide segmentation) were challenging to identify and rank according to their respective cytogenetic numbers (Figure 2A) except for a few larger ones (e.g., chromosome 1) based on their morphology (Supplementary Figure S3B,E). The indistinct centromere position that separates the p and q arms of the chromosome made it challenging to determine and identify each chromosome. After segmenting the data using narrow segmentation threshold level (minimum 38,445 maximum 41,264), a more brighter axial core on the chromosomes (Supplementary Figure S3A,D) and the morphology of all the chromosomes including their centromere positions were more evident (Figure 2B,C; Supplementary Figure S3A,D) compared to medium segmentation threshold level (minimum 37,596 maximum 43,832) (Supplementary Figure S3B,E). The centromere allowed separation of both the p and q arms of the chromosome (Figure 2D,E) and permitted the precise determination of one chromosome’s identity from the others. Ranking of chromosomes was done after plotting graphs for both volume (Figure 2F; Supplementary Table S1) and length (Figure 2G; Supplementary Table S1) of the whole chromosomes vs. the centromere indices (p arm). Chromosomes 1–3 (group A), 4–5 (group B) were easily identified with their homologs from both their length (Figure 2F) and volumes (Figure 2G). Chromosome X and Y (group H), the sex chromosomes, were distinguished from the volume graph. Chromosomes 16–18 (group E), 19–20 (group F), and 21–22 (group G) were all separated into different clusters allowing them to be distinguished from one another, whereas chromosomes 6–12 (group C) were all intermixed within one cluster, making them extremely difficult to be identified. In our analysis, group D chromosomes (13, 14, and 15) that are acrocentric were difficult to rank based on their morphology, but as a group, they were distinguishable from the rest of the karyotype. The chromosome morphology together with length and volume measurements was critical to identify the individual 46 chromosomes into their respective pairs and cytogenetic groups (Figure 2F,G).

### 2.3. Validation of Chromosomes 

**Figure 3:**
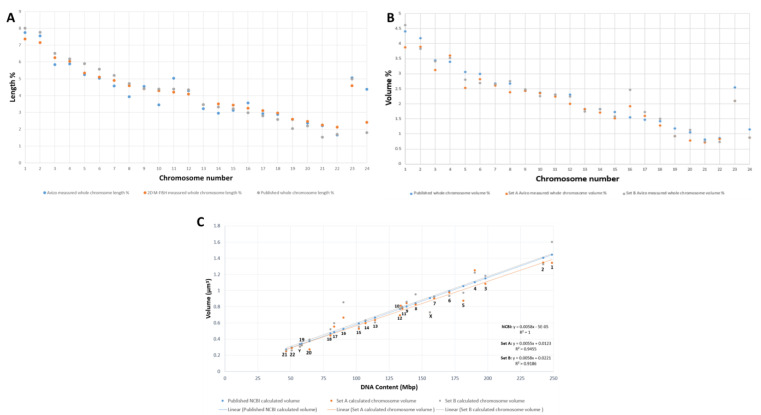
Validation parameters of 46 chromosomes after SBFSEM and Avizo segmentation. (**A**) Comparing the length of our measured chromosomes with length measurements of our mFISH 2D spread chromosomes and published chromosome length data [43]. (**B**) Comparing the volume of both homologs (set A and set B) of our measured chromosomes with published chromosome volume measurement [44]. (**C**) Comparison of published and our measured chromosome volumes of both homologs (set A and set B) versus the number of base pairs of the accordingly assigned chromosomes from the human genome sequence [3].

To validate the identified individual chromosomes, we used a multi-parametric verification strategy. The homologs of each chromosome pair (1–22) except X and Y were divided into two sets: a and b. All measured chromosomes except 10, 11, and Y from our 3D data were in agreement with lengths (p and q arms) obtained from 2D metaphase chromosome preparations from the same cell line (Figure 3A, Supplementary Figure S2F, Supplementary Table S1) and also with published length data [43] (Figure 3A). Our chromosome volume data (Supplementary Table S1) are also in agreement with early published volume measurements [44] except for chromosomes 1, 3, 5, 16, and X (Figure 3B). Comparison of the known DNA content from the human genome database (https://www.ncbi.nlm.nih.gov/genome/guide/human/ (last accessed in September 2020) and Pioveson et al. 2019 [3] with our wide segmentation chromosome volume data displayed a linear relationship for all chromosomes except 1 and 16 (Figure 3C; Supplementary Table S2). This linear relationship was also obtained from the calculated DNA content of narrow segmented chromosomes but did not correspond to the NCBI DNA content and volume data (Supplementary Figure S4A,B). All measured individual chromosomes resided in the plot based on their size ranging from larger chromosomes to smaller except for chromosome 21, which contains the least DNA (46.7 Mbp) in the human genome and falls below chromosome 22 (50.8 Mbp) in the plot. A similar arrangement was also seen for chromosome 19 (58.6 Mbp) that resided below chromosome 20 (64.4 Mbp) (Figure 3C) and agrees with published sequence data [3]. Chromosomes 9–12 were all clustered very close to one another, making them difficult to be distinguished within group C. These chromosomes are known to have similarity in bp composition, size, and GC percentage [3,44,45]. Even wider segmentation (referred to as wide segmentation)—contrast threshold minimum 37,498 and maximum 41,346) was done for four chromosomes 1b, 8b, 16a, and 18b following a wide contrast threshold (Supplementary Figure S3C,F). The measured volume and DNA content of these chromosomes were almost double the size of medium segmented chromosomes (Supplementary Figure S4C,D, Supplementary Table S3), representing a close to tetraploid (4n) chromosome DNA content. Measured diameters of the four chromosomes at three levels of segmentation—narrow, medium, and wide—were 0.63 ± 0.05, 0.91 ± 0.11, and 1.06 ± 0.07 µm, respectively (Supplementary Figure S5). This indicates the extent of chromosome compaction and also determines the overall state of the nucleus to be in late prophase. This also indicates a widespread gradation of the degree of compaction with diameter among all the chromosomes.

### 2.4. Building the 3D Human Genome

**Figure 4:**
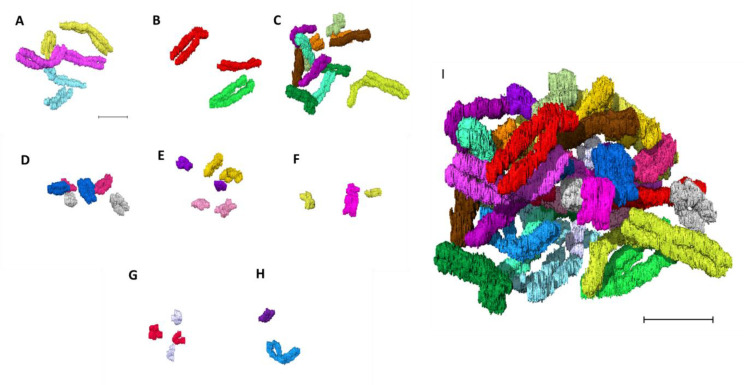
Building the 3D human genome. Chromosomes paired in the same color and classified into eight groups (**A**–**G** (autosomes) and **H** (sex chromosomes)). (**A**) group A (chromosomes 1–3), (**B**) group B (chromosomes 4 and 5), (**C**) group C (chromosomes 6–12), (**D**) group D (chromosomes 13–15), (**E**) group E (chromosomes 16–18), (**F**) group F (chromosomes 19 and 20), (**G**) group G (chromosomes 21 and 22), (**H**) group H (sex chromosomes X and Y), (**I**) 46 chromosomes making the human genome after assembling A–H. Scale bar—2 µm.

**Figure 5:**
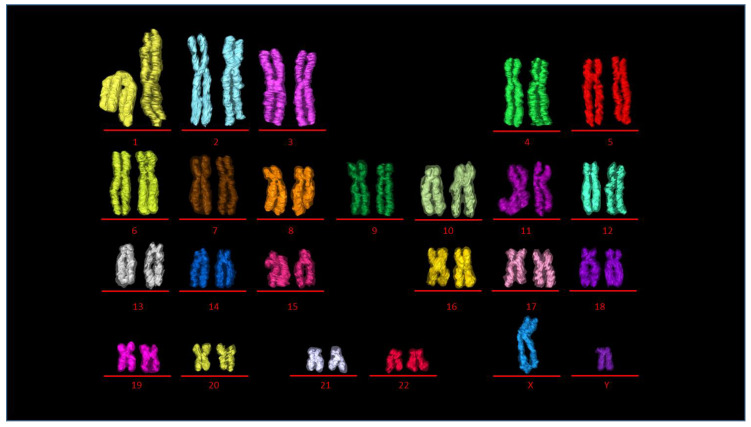
3D karyotype. 3D pseudocolored karyotype displaying 23 chromosome pairs 1–22 (autosomes), X, Y (sex chromosomes). Transparent outline is the medium segmentation and inner color is from the narrow segmented data.

Chromosomes were paired in the same pseudo-colors and classified into eight groups (Figure 4A–G (autosomes) and Figure 4H (sex chromosomes)) assembling the human genome consisting of 46 chromosomes (Figure 4I). Chromosomes from the eight groups (A) group A (chromosomes 1–3), (B) group B (chromosomes 4–5), (C) group C (chromosomes 6–12), (D) group D (chromosomes 13–15), (E) group E (16–18), (F) group F (chromosomes 19–20), (G) group G (chromosomes 21–22), and (H) group H (sex chromosomes X and Y) were organized into their respective pseudo-colored pairs in the first of its kind 3D karyotype based on their axial structure from narrow segmentation (Figure 5). This compact axial structure covers the core of the p and q arm that was seen for all the chromosomes analyzed. The chromatids for both the p and q arms of each chromosome reside in a parallel configuration with no crossover (Figure 4 and Figure 5). One chromosome 1 (Figure 4A and Figure 5) showed folding of the p arm, and one chromosome 11 (Figure 4C and Figure 5) showed folding of the q arm. Although a parallel chromatid configuration for each chromosome is seen, a curved morphology was observed for several chromosomes at different 3D rotational angles (Supplementary Figure S6). 

### 2.5. Radial Organization of Chromosomes in the Prophase Nucleus

**Figure 6:**
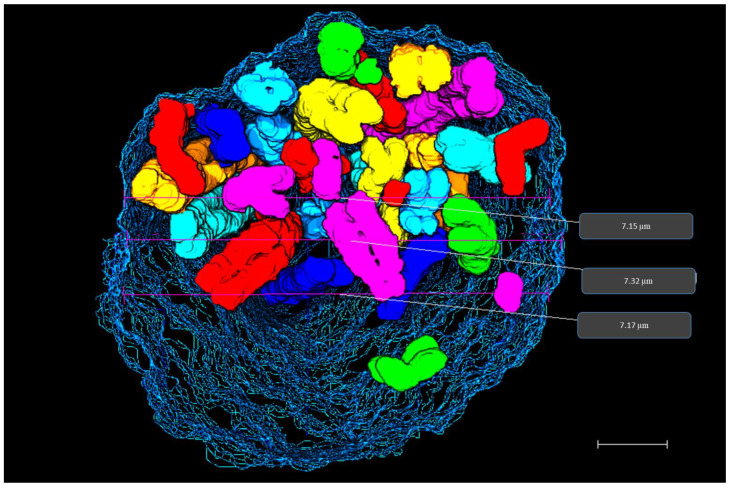
3D prophase nucleus. 3D rendering of prophase nucleus showing chromosomes enclosed within the nuclear membrane reconstructed from SBFSEM data stack. Diameter measurements of prophase nucleus are displayed on the rendered data in grey boxes. Scale bar—1 µm.

The diameter of the nucleus was measured to 7.2 µm (Figure 6). The entire data stack consisted of 345 slices taken with 25 nm sectioning, which amounts to a diameter of 8.6 µm. The 3D view shows that chromosomes occupy the internal nucleus space (Figure 6). Supplementary video S1 shows all 46 chromosomes embedded inside the prophase nuclear membrane.

**Figure 7:**
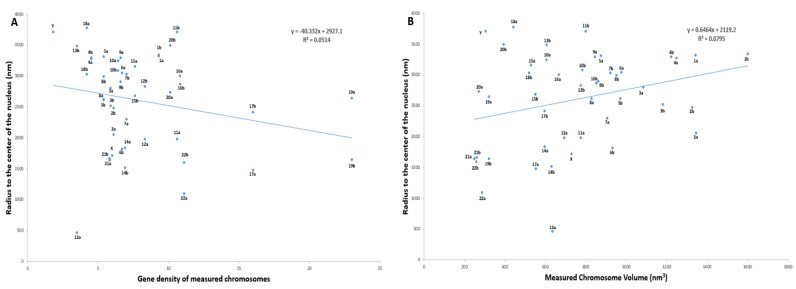
Radial organization of all 46 prophase chromosomes in a 3D nuclei. (**A**) Scatter diagram showing correlation between chromosome volume and radius to the center of the nucleus. (**B**) Scatter diagram showing correlation between chromosome gene density and radius to the center of the nucleus.

To determine the radial organization of the prophase chromosomes, we plotted the values for radial distance obtained for each medium segmented chromosome (Supplementary Table S2) with their corresponding volume and gene density separately. Regression analysis was performed to demonstrate the existing relationship between two variables and gave a weak positive correlation with R^2^ values of 0.051 (volume) and 0.080 (gene density). According to the five equally divided regions on the plots, homologs of gene-rich chromosomes 17a, 22a and gene-poor chromosome 13a occupied a more interior location within the nucleus, whereas gene-poor chromosomes 13b, 18ab, and Y were localized closer to the nuclear periphery (Figure 7A; Supplementary Figure S7A). Chromosome X was located towards the intermediate (between the center and periphery) of the nucleus. (Figure 7A; Supplementary Figure S7A). Larger chromosomes including both homologs of chromosome 1, 4 and one homolog of 5(a) were more peripherally located, whereas, chromosome 2, 3 and the other homolog of 5(b) were positioned towards the periphery-intermediate of the nucleus (Figure 7B; Supplementary Figure S7B). Although the most gene-rich chromosome, 19, occupied an intermediate position instead of the nuclear center, the preferential positioning of chromosomes Y, 18, and 20 on the immediate periphery irrespective of their volumes supported the presence of gene density-dependent correlation (Figure 7A; Supplementary Figure S7A). It was pointed out by Cremer and Cremer (2010) [1] that the appearance of the large chromosomes at the exterior of the distribution, as confirmed here, goes against statistical mechanical principles and requires an active driving mechanism.

### 2.6. 3D Chromosome Neighborhood Analysis

**Figure 8:**
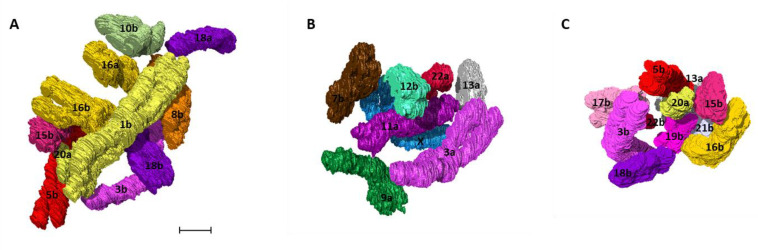
Neighborhood clusters. 3D clusters of the closest spatial neighbors for human chromosome (**A**) 1b (**B**) 11a (**C**) 19b detected by 3D analysis using Avizo. Colors of the chromosomes correspond to the pseudocolor karyotype (Figure 4). Scale bar—1 µm.

Visual analysis to map out the spatial neighbors for all 46 human prophase chromosomes was performed by observing the position of each chromosome in relation to the rest of the chromosomes in 3D nuclear space. Chromosomes present in closer proximity to an individual chromosome were characterized as its spatial neighbors (Supplementary Figure S8). As an example, chromosome 1b was closely surrounded by 10 other chromosomes including 3b, 5b, 8b, 10b, 15b, 16a, 16b, 18a, 18b, and 20a (Figure 8A). Examples for chromosome 11a (7 neighbors) and 19b (10 neighbors) with their neighboring chromosomes are given in Figure 8B,C respectively.

## 3. Discussion

The SBFSEM method is proving very useful for determining chromatin folding structures in a range of systems [46] and has been employed previously to determine chromosome morphology. The spatial arrangement of an incomplete set of chromosomes within a 3D prophase nucleus has been previously reported, but it resulted in the identification of only a limited number of chromosomes [39,40] therefore, our aim here was to identify all 46 chromosomes in a complete 3D prophase nucleus. We also aimed to determine their spatio-temporal organization at a stage of the cell cycle preceding the formation of the spindle structure and the kinetochore that leads the cell to mitosis. The number of previous studies that have examined the 3D organization of genomes mapping all 46 chromosomes is very limited. FISH studies using chromosome specific probes on lymphocytes have allowed mapping of all chromosome territories to compare the spatial relationships relative to the interior or periphery of the interphase nucleus [47,48]. This strategy has also been applied on human sperm nuclei using a multicolor banding approach [49]. Multicolor FISH has allowed mapping of all 46 chromosomes [36,37], and single cell Hi-C has also allowed genome-wide analysis of 3D chromosome interactions [38,50].

The identification of segmented chromosomes is crucial to study their 3D spatial positioning individually and in relation to each other using SBFSEM. We could not rank segmented chromosomes according to their respective numbers based on volume information only, apart from few large and small chromosomes. This is mainly due to human chromosomes having similar base pair sizes with slight variation amongst them [3]. Human chromosomes are generally identified after performing G-banding [51] or applying 24 color FISH probes on fixed metaphase spreads [52]. For electron microscopy imaging, no chromosome-specific stains are available, making chromosome identification difficult. Therefore, in this study, we applied quantitative measurements to every chromosome for length, volume, and DNA content concurrently with morphology and centromere position information to accurately identify as many chromosomes as possible. 

The prophase stage of the cell cycle is understudied. The cell cycle stage of the nucleus in this study can be recognized by examining the morphology and compaction of the chromosomes. A distinctive feature of chromosomes in late prophase is the appearance of sister chromatids as individualized, parallel arms, with no twists through the entire length of the chromosome [7,39,53]. The late prophase chromosomes display arrays of consecutive loops condensed around a central axis that get thicker as they approach prometaphase [7,15]. Similar parallel alignment of sister chromatids is evident in our 3D chromosome constructs as well. Diameter measurements of our chromosomes are consistent with Kireeva et al. 2004 [9], as they showed the condensation of prophase chromosomes following three structural transition events. Interestingly, the wide segmentation-obtained diameter (1.06 ± 0.074 µm) is indicative of the late prophase stage, in which chromosome diameters roughly double to ≈0.8–1.0 μm [9]. The radially graded density is consistent with looping models derived from Hi-C studies.

The calculated DNA content of narrow segmented chromosomes did not correspond to the NCBI DNA content and volume data. This is despite having characteristic morphology. On the other hand, the DNA content of the medium segmented chromosomes did agree. Prophase chromosomes are structurally tetraploid (4n), having double the number of base pairs than a diploid genome [54]. This required careful choice of the segmentation threshold to account correctly for the amount of DNA using medium segmentation. 

We observed that the axial core of the chromatids displayed brighter region using the narrow segmentation threshold parameters. In metaphase chromosomes, the axial structure has been seen after DNA staining [55]. The sister chromatid axes known as a ‘mini-axis’ are linked by evenly spaced bridges (stable inter-sister linkages) [6] and are further composed of scaffold proteins [11] namely condensin and Topo Iiα, which are essential for maintaining chromosome structure. Our microscopy data are in agreement with previous findings where a model has been proposed of chromatin forming sequential loops along the axial structure during prophase using Hi-C [15]. Our data show the axial structure assembly present on all late prophase chromosomes, as seen by Hi-C [15]. In our study, it is interesting to find that the DNA axial structure holds enough structural information, allowing the building of a 3D karyotype at late prophase even before the complete condensation into mitotic chromosomes. Typical mitotic morphologies are seen whereby the centromere positions could be resolved. This allowed reliable ranking in their respective cytogenetic groups, including the group C chromosomes that are generally difficult to rank even after solid staining on glass slides or during flow karyotyping [51]. Interestingly, the compact axial structure covers the chromosome arm, including the centromeres but it remains to be tested which sequences reside within the axial region of the chromatids.

Chromosomes within the prophase nucleus in this study matched a gene density-based radial organization. This is in agreement with published studies on lymphocytes using microscopy [37,47,56,57,58] performed at the interphase stage of the cell cycle. At prophase, a gene density correlation has also been observed after positioning 36 chromosomes in a partially analyzed nucleus [39]. Our study indicates that this chromosomal organization pattern of gene density is conserved from interphase to prophase [39], with certain exceptions. For example, the gene-rich chromosome 19 has been shown to occupy an interior position during interphase [56,57,58], whereas in our study, both chromosome 19s were found to occupy more intermediate positions within the prophase nucleus. These changes may be attributable to the explicit movement of chromosomes during late prophase in preparation for alignment on the metaphase plate that might represent a step toward the territory breakdown. Even though the territories are known to remain stable throughout interphase and break down at prometaphase, they reestablish in G1 [1,59] but with a different neighborhood pattern [60,61]. Our study not only gives the organization of all 46 chromosomes but also provides the first neighborhood map of the relative positioning of all chromosomes within the prophase nucleus. 

However, the useful SBFSEM approach comes with several limitations. This includes (i) the lack of haplotype determination from the maternal and paternal alleles as all chromosomes displayed with the same contrast, (ii) the correct characterization of complex chromosomes with more than 46 chromosomes, i.e., cancer genomes, (iii) any samples that are fixed in harsh chemicals having artifactual effects, and (iv) the lack of validation, as no electron microscopy probes (chromosome paints) are available.

Further studies are required to look for differences in the proximity patterns of chromosomes in different (donor) and diseased cell types, including different tissues and organs. It is yet to be established if this arrangement is affected by cell identity, nuclear shape, surrounding cell types, and the geometry of the tissue [62]. To ensure reproducibility and to rule out heterogeneity, repeated experiments on several nuclei from different cell types are needed to see if the chromosomal organization with its neighbors and the axial structure is preserved at prophase. This will also be useful to study different single cells of the same population. The future holds great excitement for unraveling the mysterious prophase chromosomes. There is no doubt that technologies such as SBFSEM together with super-resolution microscopy and Hi-C are helpful for building precise 3D genome maps of single cells. The identification of repositioned chromosomes has the potential for being a powerful tool for examining genomes for both research purposes and clinical diagnostics.

## 4. Materials and Methods

### 4.1. Cell Culture and Nuclei Preparation

Cells were grown, and nuclei were prepared according to a previously published protocol [63,64]. A B-lymphocyte cell line (GM18507) was selected as it has been previously characterized by sequencing in the International HapMap Project [41]. Briefly, the cell line (passage 4) was cultured at 37 °C in a 5% CO_2_ atmosphere using RPMI medium supplemented with 100 U/mL penicillin and 100 µg/mL streptomycin (Gibco Life Technologies, Paisley, UK), 20% FBS (Sigma-Aldrich, Dorset, UK), and 1% L-glutamine (Sigma-Aldrich, Dorset, UK). Thymidine (2 mM) (Sigma-Aldrich, Dorset, UK) synchronization was done for 16 h. Then, cells were treated using colcemid (0.2 µg/mL (Gibco Life Technologies, Paisley, UK) for 5 h followed by KCL (0.075 M); (VWR BDH Prolabo, Dublin, Ireland) treatment at 37 °C for 5 min. Samples were fixed in Carnoy’s solution (3:1 methanol/acetic acid) and stored at 4 °C until further use.

### 4.2. Multicolor Fluorescence In Situ Hybridization 

Multicolor fluorescence in situ hybridization (mFISH) was performed using a 24Xcyte mFISH probe kit according to the manufacturer’s instructions (MetaSystems, Altlussheim Germany) as well as previously published protocols [52,65,66]. Briefly, slides were washed in 100% ethanol for 1 min and then left to air dry. The sample was denatured in NaOH and subjected to ethanol dehydration (30%, 70%, 90%, and 100%) followed by air drying. After denaturing the mFISH probe at 75 °C for 5 min (reanneal incubation at 37 °C for 30 min), 10 μL of the probe was placed onto the denatured slide. A coverslip was placed over the slide followed by sealing the edges of the coverslip using rubber cement. The slides were kept in a humidified hybridization chamber at 37 °C for at least 72 h. Washing of the unbound probe was done by keeping the slides in prewarmed (72 °C (±1 °C) 0.4× SSC for 2 min. The slides were further incubated into the coplin containing 2× SSC with Tween 20 for 30 s. Finally, the slides were washed with Milli-Q water to avoid crystal formation and left to dry in air at room temperature. The probe consists of 24 painting probes specific for the 24 different human chromosomes. Each probe is labeled with up to five different fluorophores in a combinatorial labeling format to provide 24 distinct colors. Probe-labeled chromosome spreads were visualized using a Z2 Zeiss fluorescence microscope. Then, mFISH images were analyzed using the ISIS software from MetaSystems.

### 4.3. Sample Fixation, Staining, and Embedding for SBFSEM

Samples were prepared for SBFSEM based on the previously published protocols [39,67]. Briefly, the sample was fixed using 2.5% (*v*/*v*) glutaraldehyde (Sigma-Aldrich, Dorset, UK) in 0.1 M cacodylate buffer (pH 7.2) for 1 h at room temperature. Heavy metal platinum blue staining (5 mM) was done for 30 min at room temperature, followed by 2 Milli-Q water washes. The sample was subjected to gradual dehydration using ethanol–water solutions (30, 50, 75, and 100%) (Fisher Scientific, Loughborough, UK) for 15 min each. All of the above steps were performed in a 1.5-mL microcentrifuge tube, and tubes were centrifuged at 1750× *g* for 10 min before and after the change of solution. The sample was embedded in Agar 100 resin (Hard) (Elektron Technology, Cambridge, UK), and the two-step protocol for embedding was applied. In the first step, the small amount of resin was cured overnight at 60 °C, followed by the second step that involved layering of fresh resin and another round of curing for 16 h to achieve solidification. 

### 4.4. SBFSEM Sample Block Preparation and Imaging 

Samples were prepared for SBFSEM [68] and based on the previously published protocols [39,67]. To prepare the pyramid-shaped block with an upper face of 500 μm × 500 μm, the cured sample was trimmed using a conventional ultramicrotome (Leica Ultracut UCT; Leica, Buffalo Grove, IL) after mechanical polishing. Then, 25 nm serial sectioning (horizontal) was performed using an in-built diamond knife in the SBFSEM system (FEI Quanta 250 field-emission gun environmental SEM (FEGESEM)) using a cutting speed of 0.3 mm/s. Imaging using electrons was performed at 5 kV under a chamber pressure of 60 Pa of water vapor. To get full coverage of the entire single nucleus, the pixel size of the backscattered electron (BSE) micrographs was set to 11 nm × 11 nm per slice with a field of view of 22.5 × 22.5 μm. Overall, 345 slices were analyzed for the single prophase nucleus. 

### 4.5. 3D Reconstruction and Modeling of Prophase Chromosomes

Raw Tiff images obtained from SBFSEM were converted into 16 bit using Image J software (https://imagej.nih.gov/ij/download.html, accessed in September 2020). Electron microscopy (EM) data stacks were annotated using Avizo (FEI-ThermoFisher Scienctific). Using Avizo features, the data were cropped using the crop tool to display the region of interest (ROI) (nuclei) by using an extract sub-volume feature. A bilateral filter in the *x-y* planes to remove possible pixel noise was applied, which was followed by specific contrast thresholds. For the segmentation of chromosomes from a single prophase nucleus, three different threshold levels were used: (1) narrow segmentation—minimum 38,445, maximum 41,264; (2) medium segmentation—minimum 37,596, maximum 43,832; and (3) wide segmentation—minimum 37,498, maximum 41,346. Chromosomes present in every orthoslice (using xy, xz, and yz) were segmented (manually) using a combination of brush and magic wand tools with the use of interpolation when required. Nuclear membrane was also built using the brush tool (manually). 

### 4.6. Identification and Validation of Chromosomes

The identification of each segmented chromosome was made by quantitative analysis of their length, volume, and DNA content. The measurements obtained were subjected to several absolute and comparative analyses to ascertain the correct identity of all chromosomes.

Length measurements were done for individual chromosomes using the “measure” tool (line) in Avizo project view. For each chromosome, length measurements (nm) were obtained for p and q arms separately by using the line tool from one end (shorter) of the chromosome toward the centromere and from the centromere to the other (longer) end of the chromosome respectively. The centromere for each chromosome was identified as a structure that separates the two chromosome arms (p and q). Then, the measurements for both p and q arms were added up to obtain the length of the whole chromosome. For further analysis, the entire lengths of all chromosomes were converted into percentages (concerning whole genome) by dividing the length of the individual chromosome by the total length of all chromosomes multiplied by 100. Similar calculations were done to obtain the percentages of p and q arms that make up a whole chromosome (Supplementary calculation S2). The centromere index calculated the length/volume of the short arm (p) as a percentage of the chromosome’s total length/volume. Length measurements (p and q arms) were also performed on 5 mFISH spreads from the same cell line (GM18507) selected randomly. These measurements were taken in ISIS software (Metasystems) using the length tool. Mean lengths were calculated for p arms and whole chromosomes that were then converted into percentages (Supplementary calculation S3). 

Volume measurements (nm^3^) for individual chromosomes were extracted from the Avizo “Material statistics” module. Volume measurements of p and q arms were measured individually for all 46 chromosomes. For this purpose, the p arm of each chromosome was dissected out using the “Lasso” tool in the Avizo selection panel. The volume of the q arm was extrapolated from “material statistics”, and the volume of the p arm was calculated (total volume of chromosome—q arm volume = p arm volume). Volume measurements for whole chromosomes, including both p and q arms, were converted into percentages (Supplementary calculation S1).

The DNA content for each chromosome was calculated as described by Chen et al. 2017 [39]. As per published calculations, the total volume of chromosomal protein–DNA complex is expected to be 5.80 nm^3^/bp. Therefore, the volume of each chromosome was divided by 5.80 nm^3^/bp to get the number of base pairs present in a given chromosome. Base pairs calculated for individual chromosomes were converted into Mbp by dividing by 1000,000 (1 Mbp = 1,000,000 bp) (Supplementary calculation S4).

### 4.7. Diameter Measurements of Chromosomes 

Diameter measurements were extracted for a selected number of chromosomes using the “measure” tool in Avizo software (Supplementary calculation S5). 

### 4.8. Assessment of 3D Spatial Positioning of Chromosomes

Radial positioning of chromosomes within the nucleus was assessed by calculating their distance from the center of the nucleus mass (Supplementary calculation S6). Center (X, Y, Z) coordinates were extracted for each chromosome and for the nuclear boundary from Avizo using the “material statistics” module. The center of the nucleus was taken from the spatial center from Avizo. The resulting radius values, the distance from this center, were obtained for each chromosome and plotted against their measured volume and also gene density [69] to determine the spatial relationship of chromosomes within the prophase nucleus. Plots were divided into five equally spaced radial regions representing periphery, periphery–intermediate, intermediate, intermediate–center, and center of the nucleus. To identify the spatial neighbors for each chromosome, a heat map/interaction map was built.

## 5. Conclusions

To date, the processes involved in chromatin condensation into a single chromosome are not fully understood, including chromosomal positioning during the cell cycle. Given the importance of correct chromosome identification to study their spatial organization in 3D, this represents the first study to visualize all 46 intact chromosomes in a human prophase nucleus successfully. Our SBFSEM images allowed building a single-cell fingerprint of identified chromosomes in 3D nuclear space. This work gives a comprehensive view of the overall structure and organization of a human prophase nucleus. Identification of all 46 intact chromosomes and revelation of their morphologies from the axial core provides new insight into the modeling techniques for detailed inspection of chromosome organizational architecture in 3D. Chromosomes within the prophase lymphocyte nucleus were found to follow closely a gene density-based radial organization.

## Data Availability

Not applicable.

## Supplementary Materials

The following are available online at https://www.mdpi.com/article/10.3390/ijms22115987/s1, Figure S1. Segmentation of a single prophase chromosome, Figure S2. Twenty-four color karyotyping of GM18507 cell line (passage 4), Figure S3. Three levels of chromosome segmentation, Figure S4. Validation of segmented chromosomes, Figure S5. Diameter of chromosomes, Figure S6. Morphology of individual chromosomes, Figure S7. Radial organization of all 46 prophase chromosomes in a 3D nuclei, Figure S8. Spatial neighborhood, Table S1. Measurements of chromosomes after narrow segmentation, Table S2. Medium segmentation of chromosomes, Table S3. Wide segmentation of chromosomes, Calculation S1. Volume Measurement in 3D, Calculation S2. Length Measurement in 3D, Calculation S3. Length Measurement in 2D (mFISH spreads), Calculation S4. Calculation for DNA content of chromosomes, Calculation S5. Diameter measurement of chromosomes, Calculation S6. Calculation for finding center of chromosome position, Video S1. 3D visualization of a single prophase nucleus.

## Abbreviations

Base pairs (bp)
Chromosomal territories (CT)
Chromosome conformation capture (3C)
Electron microscopy (EM)
Fluorescence in situ hybridization (FISH)
Mega base pairs (Mbp)
Multicolor fluorescence *in-situ* hybridization (mFISH)
Nanometer (nm)
Serial block-face scanning electron microscopy (SBFSEM)
Three dimensional (3D)
Topologically associating domains (TADS)
