# Supplementary material for: Ultra-Structural Imaging Provides 3D Organization of 46 Chromosomes of a Human Lymphocyte Prophase Nucleus
Source: Int J Mol Sci. 2021 Jun 1;22(11):5987. doi: 10.3390/ijms22115987 (PMC8198510; doi:10.3390/ijms22115987)
Supplement: Supplementary file 1 [file ijms-22-05987-s001.zip › edited Supplementary/Supplementary Figures online.pdf]

Supplementary Figures

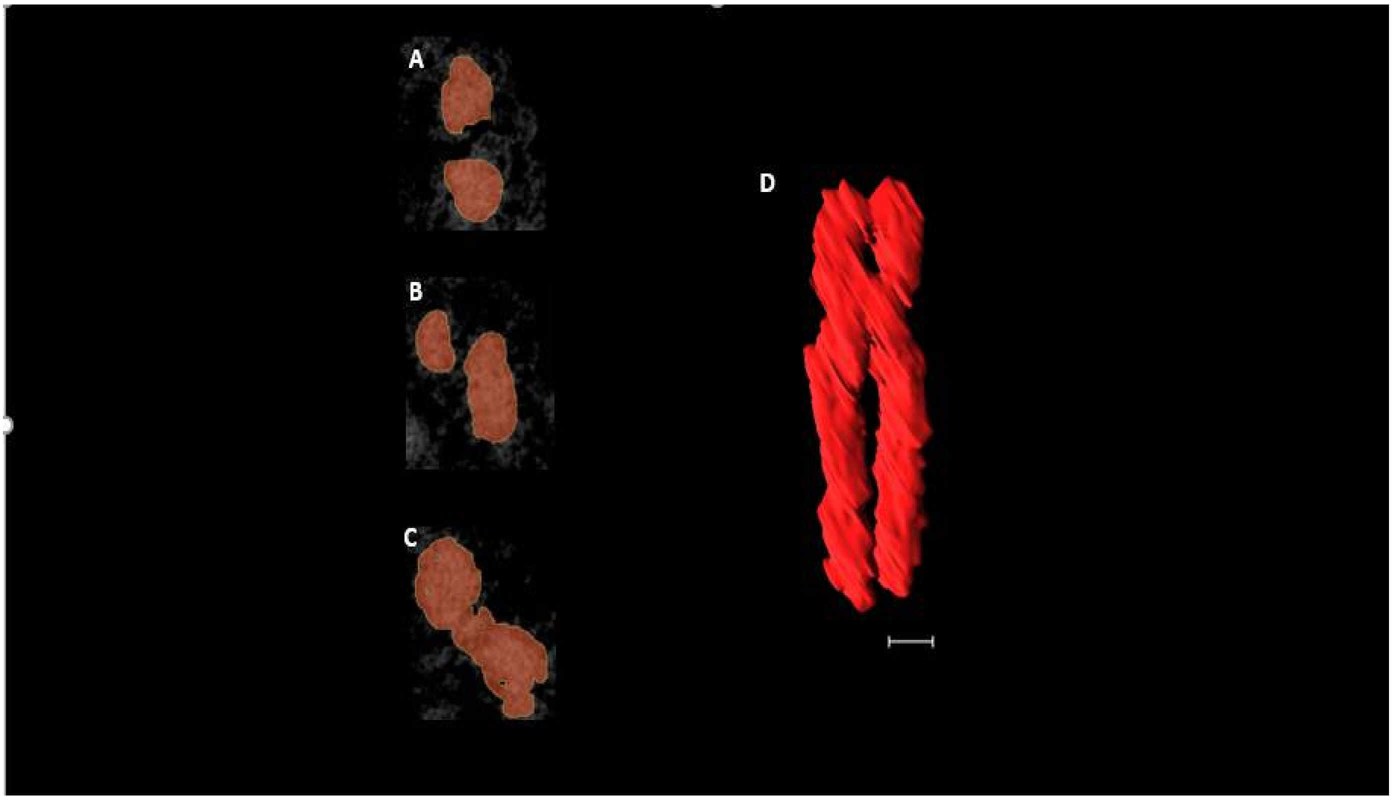

Supplementary Figure S1. Segmentation of a single prophase chromosome. (**A**) Slice no. 23 after SBFSEM of a whole chromosome. (**B**) Slice no. 39, and (**C**) Slice no. 72 which is further down the series of the same chromosome shown in (**A**). (**D**) 3D modelled and segmented image showing X-shaped human chromosome after reconstruction of 387 × 25-nm sections. Scale bar - 0.5 μM

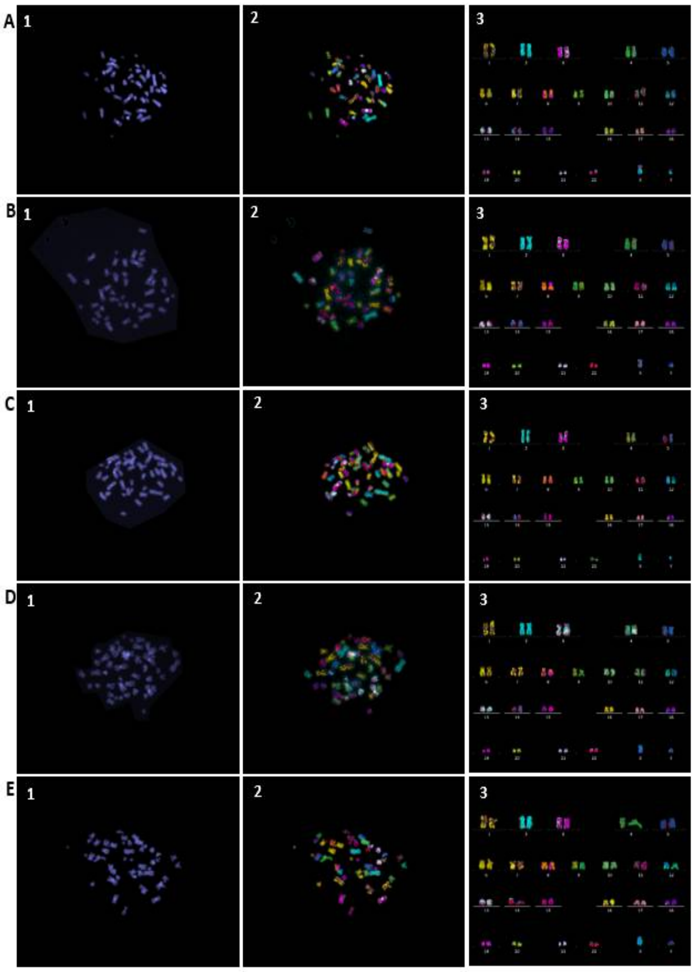

F

Chromosome no.	Averaged length (µm) (spread 1)	Averaged length (µm) spread 2	Averaged length (µm) spread 3	Averaged length (µm) spread 4	Averaged length (µm) spread 5	Total Averaged length of 5 spreads	Whole chromosome length % of 5 spreads
1	4.8	4.8	5.25	4.95	5.25	5.01	7.37
2	4.65	4.65	5.3	4.95	4.75	4.86	7.15
3	4.1	4.1	4.6	4.1	4.35	4.25	6.25
4	3.9	4	4.2	4.2	4.25	4.11	6.04
5	3.5	3.45	3.8	3.75	3.65	3.63	5.34
6	3.3	3.4	3.65	3.8	3.2	3.47	5.1
7	3.35	3.35	3.3	3.4	3.25	3.33	4.9
8	3	3.1	3	3.2	3.3	3.12	4.59
9	2.9	3	2.75	3.3	3	2.99	4.4
10	2.8	2.75	2.9	3.1	3.05	2.92	4.29
11	2.8	2.7	2.8	3	3	2.86	4.2
12	2.75	2.35	3.15	2.95	2.7	2.78	4.09
13	2.5	2.4	2.65	2.2	2.05	2.36	3.47
14	2.4	2.5	2.8	2.1	2.1	2.38	3.5
15	2.4	2.4	2.7	2.2	2	2.34	3.44
16	2.2	1.7	2.65	2.25	2.25	2.21	3.25
17	2.1	1.9	2.3	2.2	2.05	2.11	3.1
18	1.95	2.15	2.1	2	1.9	2.02	2.97
19	1.65	1.7	1.95	1.8	1.7	1.76	2.59
20	1.7	1.2	1.9	1.9	1.7	1.68	2.47
21	1.6	1.6	1.4	1.7	1.4	1.54	2.26
22	1.2	1.75	1.4	1.55	1.35	1.45	2.13
23 (X)	3	3.4	3	3.1	3.1	3.12	4.59
24 (y)	1.8	1.7	1.6	1.6	1.5	1.64	2.41
**Total**						**67.94**	**99.9**

Supplementary Figure S2. **Twenty-four color karyotyping of GM18507 cell line (passage 4)** (**A–E**) mFISH spread 1-5, 1) DAPI spread of prepared chromosomes 2) mFISH image of the same spread as shown in 1. 3) Karyotyping of the chromosomes in 2) based on color displaying 46 chromosomes. (**F**) Summary table showing length measurements of chromosomes from each spread. Calculations for Total averaged length and whole chromosome length % are given in Supplementary calculations S3.

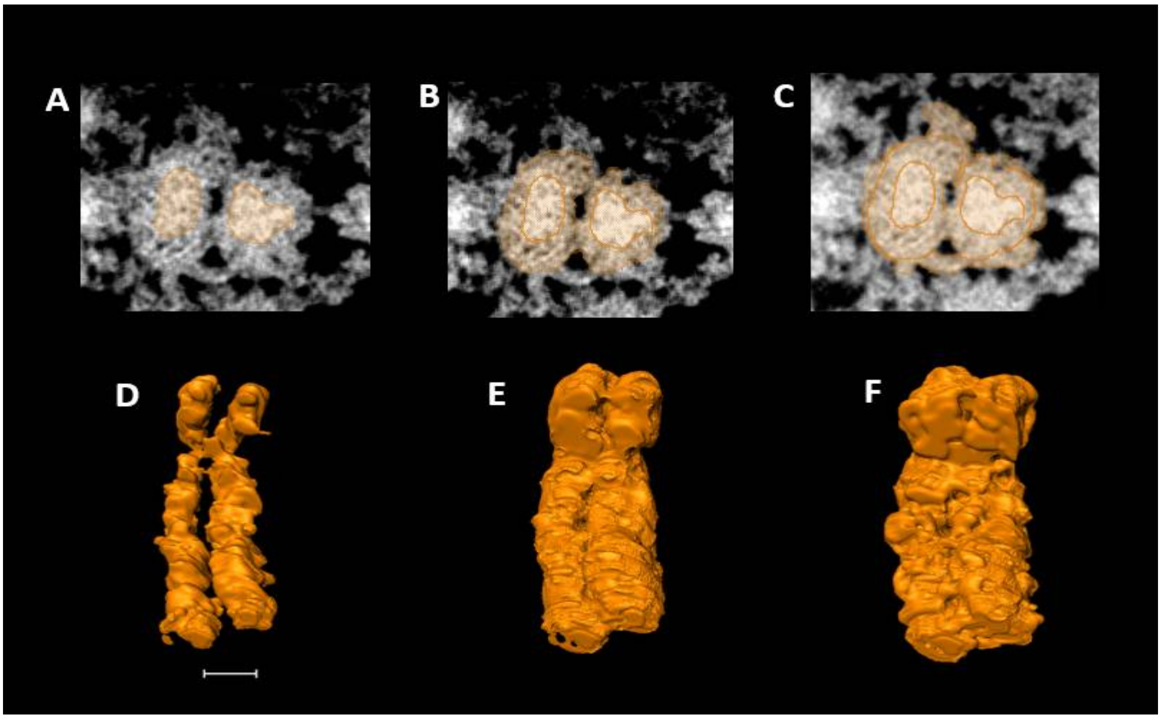

Supplementary Figure S3. Three levels of chromosome segmentation. (**A–C**) 2D segmentation of chromosome 8b following three different contrast thresholds. (**D–F**) 3D view of chromosome 8b after segmentation; (**A**) and (**D**) Narrow segmentation, (**B**) and (**E**) Medium segmentation and (**C**) and (**F**) Wide segmentation. Scale bar – 0.5 μm.

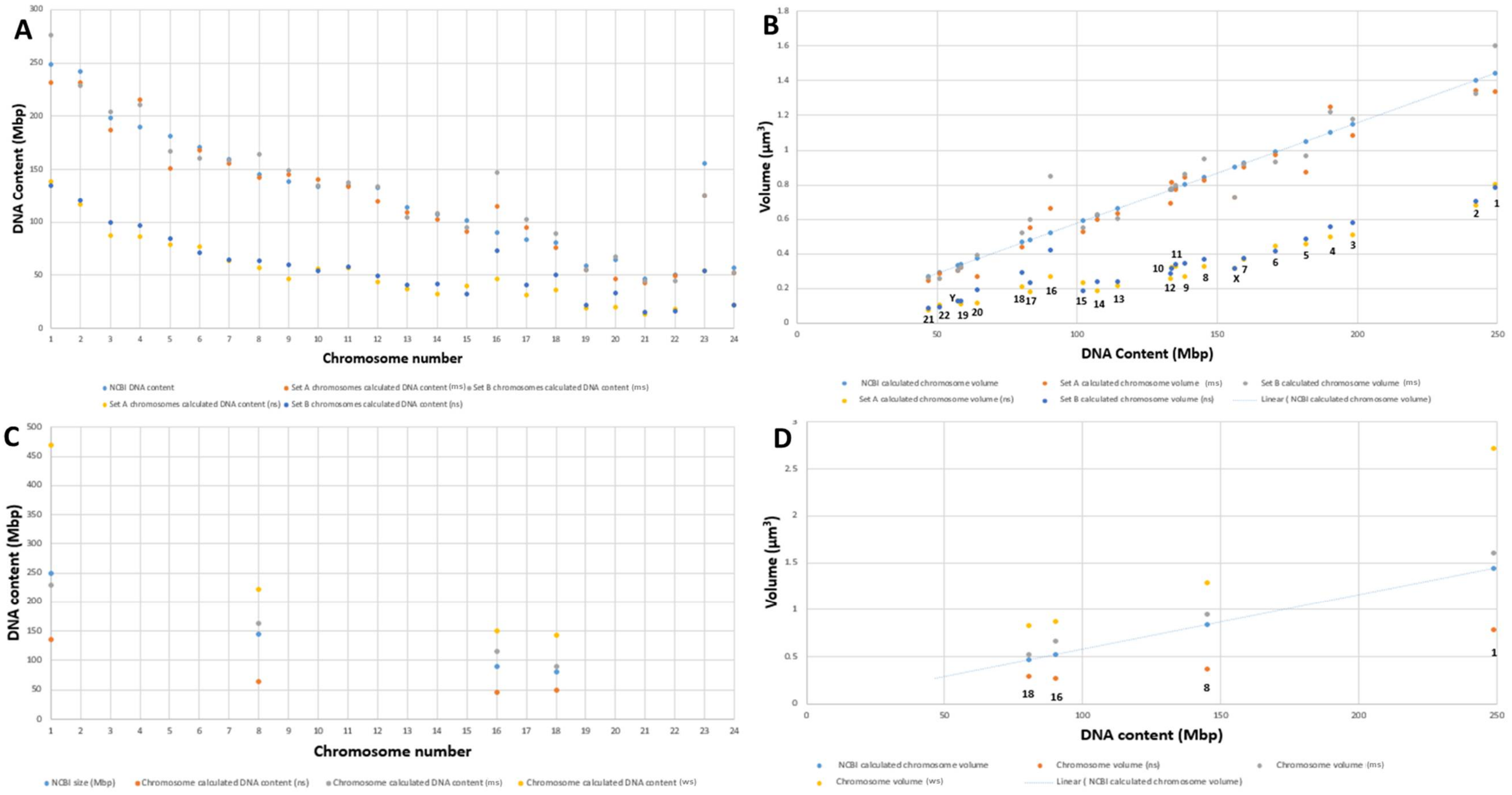

Supplementary Figure S4. Validation of segmented chromosomes. Comparison of published and our measured narrow segmentation (ns) and medium segmentation (ms) chromosome (**A**) volume and (**B**) DNA content for both homologs (set A and set B). Comparison of published and our measured narrow segmentation (ns), medium segmentation (ms) and wide segmentation (ws) C) volume and D) DNA content for chromosomes 1b, 8b, 16a and 18b. Volume and DNA content data for each chromosome is given in Supplementary Table S1, S2 and S3

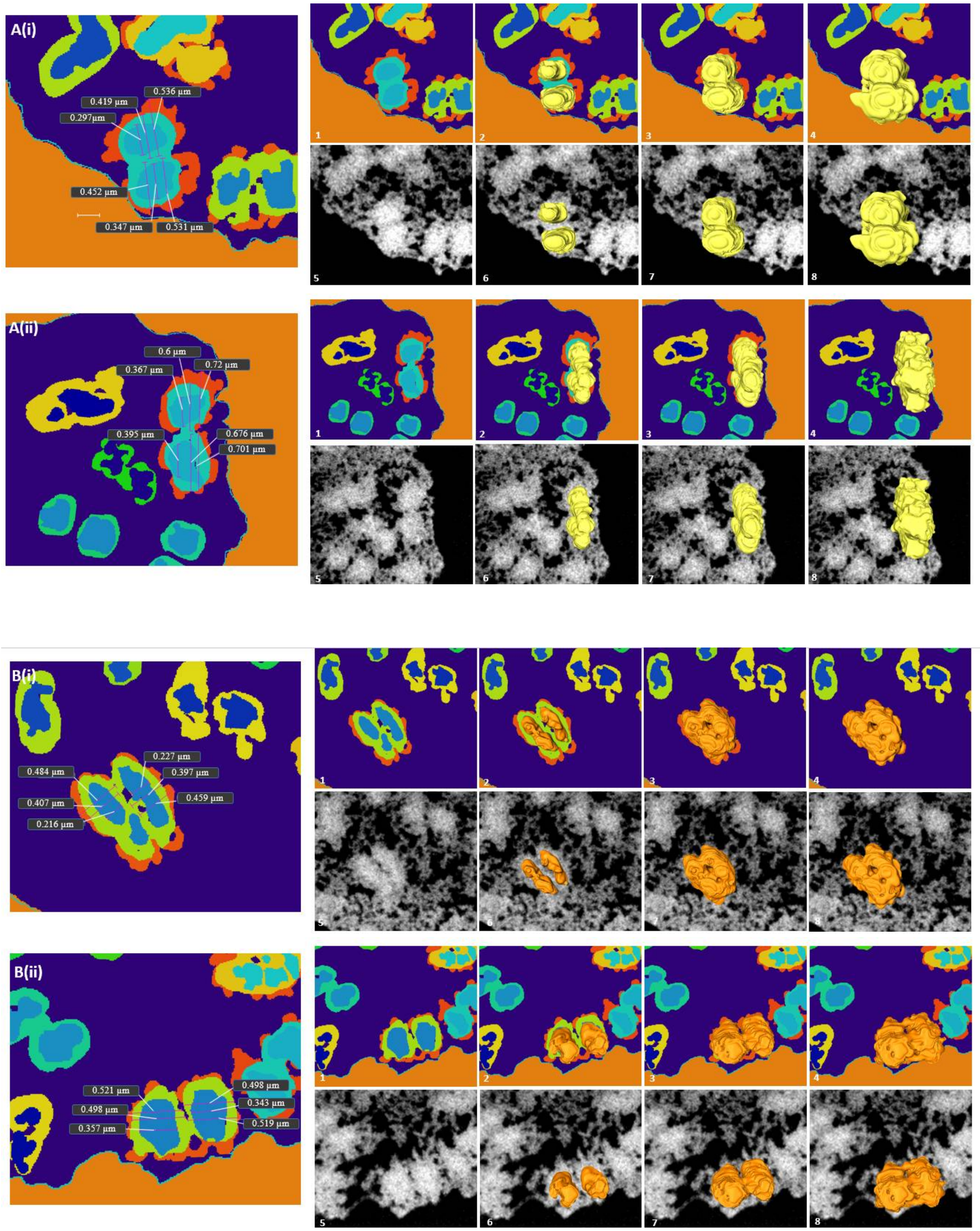
A(i)
0.419 µm
0.536 µm
0.297µm
0.452 µm
0.347 µm
0.531 µm
A(ii)
0.6 µm
0.367 µm
0.72 µm
0.395 µm
0.676 µm
0.701 µm
B(i)
0.227 µm
0.484 µm
0.397 µm
0.407 µm
0.459 µm
0.216 µm
B(ii)
0.521 µm
0.498 µm
0.498 µm
0.343 µm
0.357 µm
0.519 µm

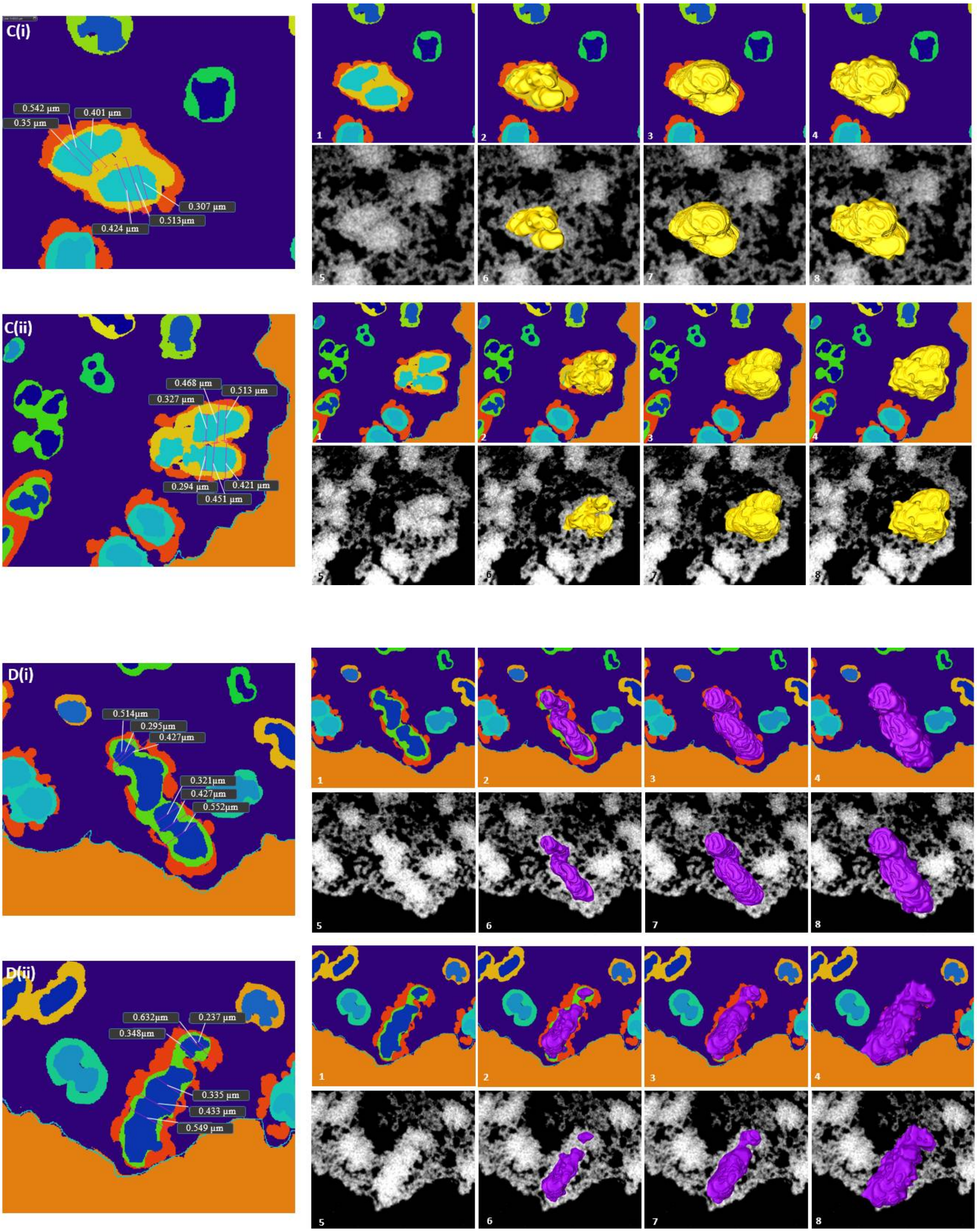
C(i)
0.542 µm
0.401 µm
0.35 µm
0.307 µm
0.513µm
0.424 µm
C(ii)
0.468 µm
0.513 µm
0.327 µm
0.294 µm
0.421 µm
0.451 µm
D(i)
0.514µm
0.295µm
0.427µm
0.321µm
0.427µm
0.552µm
D(ii)
0.632µm
0.237 µm
0.348µm
0.335 µm
0.433 µm
0.549 µm

E	Chromosome Diameters (µm)				
	Chromosome 1b	Chromosome 8b	Chromosome 16a	Chromosome 18b	Mean
Narrow segmentation	0.703	0.5715	0.639	0.594	0.63±0.057
Medium segmentation	1.0735	0.9	0.857	0.8175	0.91±0.112
Wide segmentation	1.1335	0.9915	1.0095	1.1235	1.06±0.074

Supplementary Figure S5. Diameter of chromosomes. Images (from Avizo) for diameter measurements of narrow, medium and wide segmented chromosome. **A(i)** 1b p arm and **A(ii)** 1b q arm. **B(i)** 8b p arm and **B(ii)** 8b q arm. **C(i)** 16a p arm and **C(ii)**16a q arm. **D(i)** 18b p arm and **D(ii)** 18b q arm. Image 1 and 5; orthoslice showing 2D segmented chromosome, 2 and 6; narrow segmentation 3D view, 3 and 7; medium segmentation 3D view; 4 and 8 wide segmentation 3D view. Scale bar - 2 µm (**E**) Summary table containing diameter measurements of chromosomes.

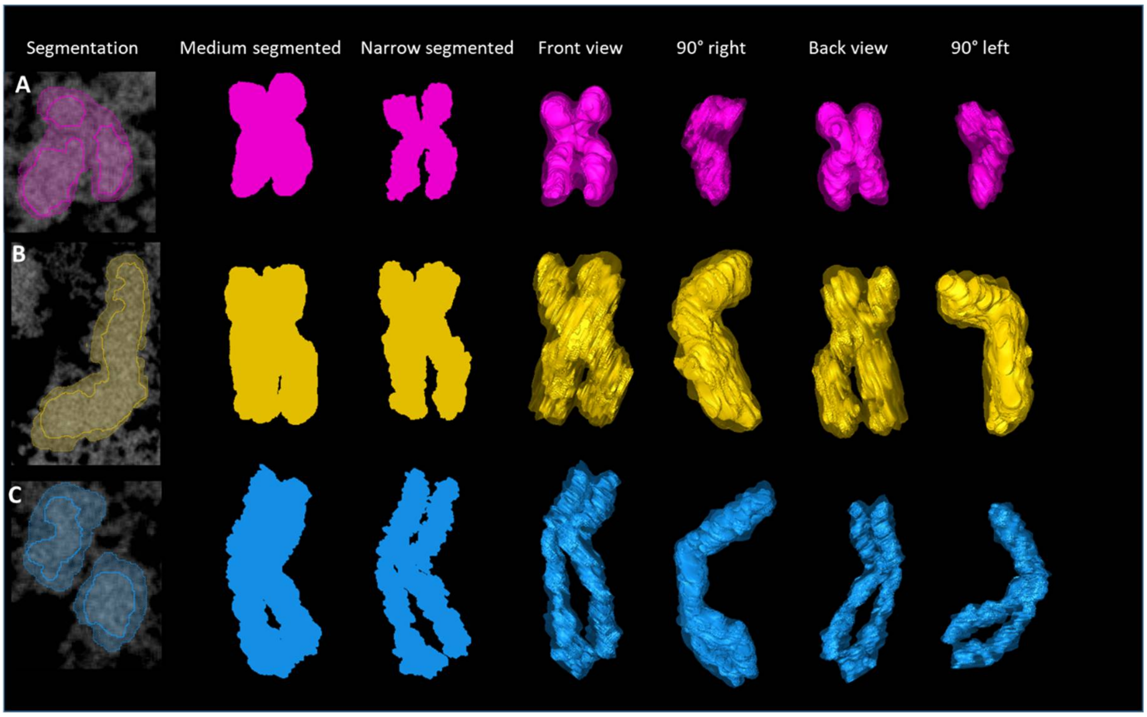

Supplementary Figure S6. Morphology of individual chromosomes. Example of 3 different chromosomes (**A**) chromosome 19 (**B**) chromosome 16 (**C**) chromosome X showing segmentation (narrow and medium) and morphology at different rotation angles.

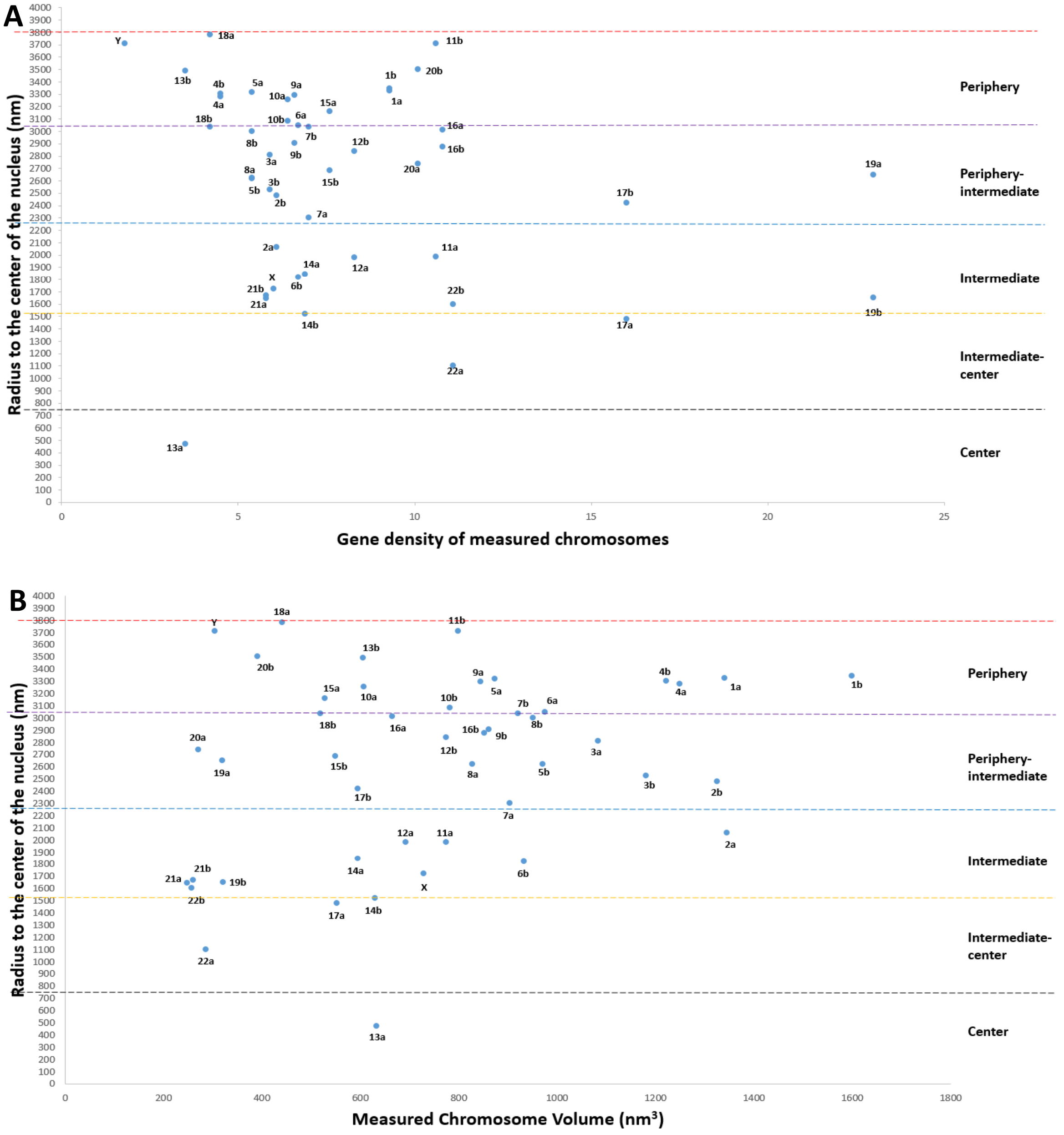

Supplementary Figure S7. Radial organization of all 46 prophase chromosomes in a 3D nuclei. (**A**) Scatter diagram showing correlation between chromosome volume and radius to the center of the nucleus (**B**) scatter diagram showing correlation between chromosome gene density and radius to the center of the nucleus. Plots are divided into 5 equal regions Periphery, Periphery-intermediate, intermediate, intermediate center and center to determine their position within the nucleus.

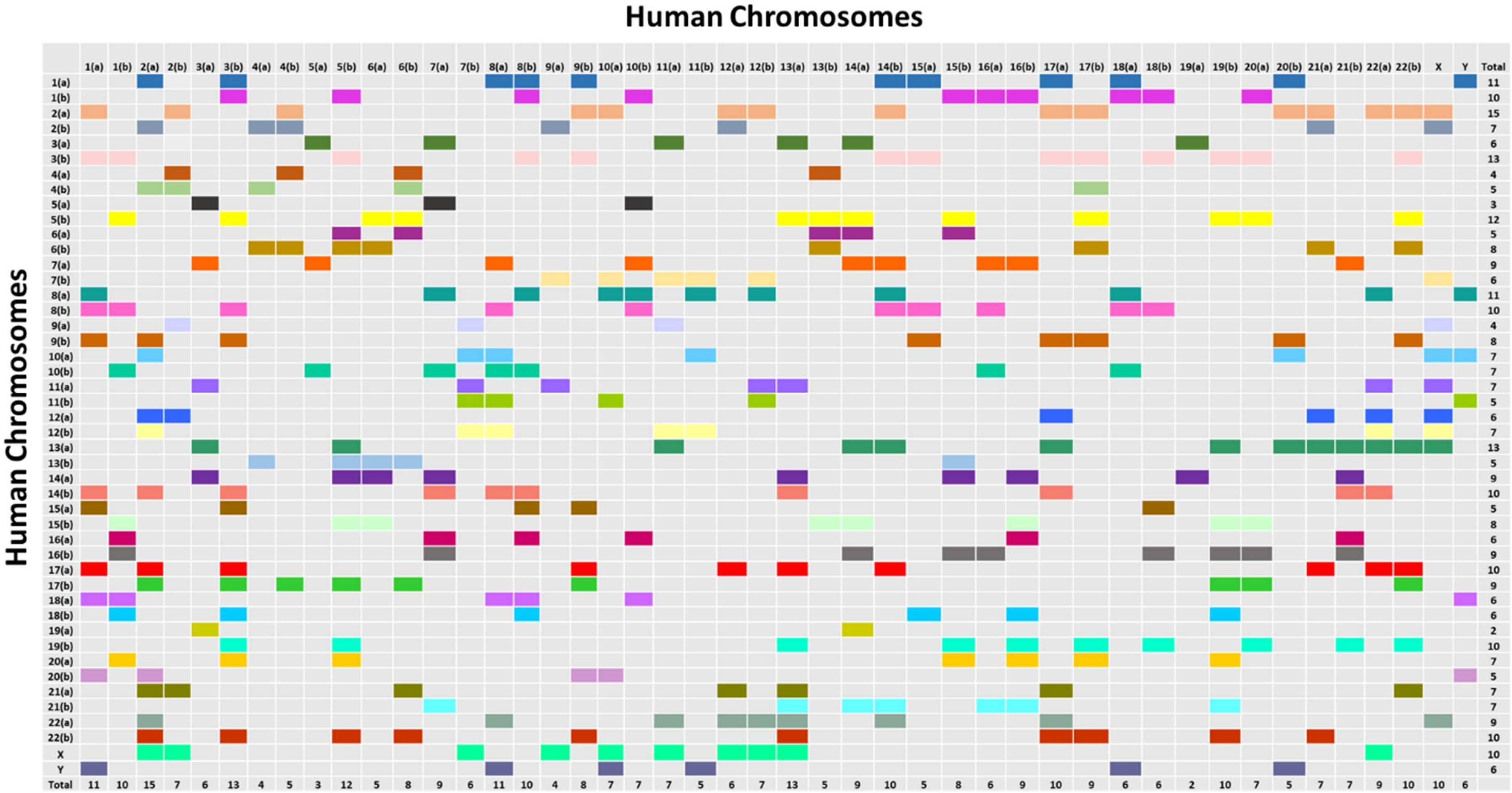

Supplementary Figure S8. Spatial neighborhood. Map of the spatial neighbors for all human prophase chromosomes detected by 3D analysis using Avizo. Filled color boxes indicate the closest neighbors of each of the human chromosomes in 3D.
