# Supplementary material for: Ultra-Structural Imaging Provides 3D Organization of 46 Chromosomes of a Human Lymphocyte Prophase Nucleus
Source: Int J Mol Sci. 2021 Jun 1;22(11):5987. doi: 10.3390/ijms22115987 (PMC8198510; doi:10.3390/ijms22115987)
Supplement: Supplementary file 1 [file ijms-22-05987-s001.zip › edited Supplementary/Supplementary Tables online.pdf]

Supplementary Tables

Material number	Resolved Chromosome number	Chromosome Volume (nm^3)	p arm volume (nm^3)	q arm volume (nm^3)	p arm volume %	q arm volume %	Whole chromosome volume %	Chromosome Length (nm)	P arm length (nm)	q arm length (nm)	p arm length %	q arm length %	Whole chromosome length %	Calculated DNA content (Mbp)
chromosome_1_-_4_final	1a	803642675	345890600	457752075	43.0	56.96	5.3	4732.68	2459.39	2571.02	47.17	52.83	3.94	138.559
chromosome_1_-_42_final	1b	784022525	364984400	419038125	46.6	53.448	5.2	5030.41	2232.66	2500.02	48.9	51.1	4.19	135.1762
chromosome_2_-_34_final	2a	677657475	200439525	501378625	41.8	58.203	4.7	4769	1817.95	2923.96	38.73	61.27	3.97	116.8374
chromosome_2_-_22_fianl	2b	701818150	283245875	394411600	28.6	71.44	4.5	4741.91	1847.14	2921.86	38.33	61.67	3.95	121.0031
chromosome_3_-_29_final	3a	508193950	251704200	327906975	44.5	55.531	3.8	3439.11	1776.67	2140.31	44.26	55.74	2.86	87.6196
chromosome_3_-_17_final	3b	579611175	157372600	401913600	43.4	56.574	3.7	3916.98	1522.22	1916.89	45.35	54.65	3.26	99.9329
chromosome4_-_20_final	4a	499947800	225991700	282202250	23.3	76.718	3.4	3661.56	1307.38	2445.61	28.25	71.75	3.05	86.1978
chromosome_4_-_16_final	4b	559286200	116398975	383548825	28.1	71.862	3.3	3752.99	1034.58	2626.98	34.83	65.17	3.12	96.4286
chromosome_5_30_final	5a	455217125	130147600	357509625	25.5	74.512	3.2	3122.33	978.26	2505.18	26.39	73.6	2.6	78.4857
chromosome_5_-_18_final	5b	487657225	116029925	339187200	26.7	73.312	3.0	3483.44	824	2298.33	28.08	71.91	2.9	84.0788
chromosome_6_-_15_final	6a	446786450	172031750	274754700	38.5	61.496	3.0	3469.08	1482.34	1986.74	42.73	57.27	2.88	77.0321
chromosome_6_-_23_final	6b	415807425	149413825	273230100	28.0	71.966	2.8	2869.49	1160.15	1709.34	40.43	59.57	2.39	71.6909
chromosome_7_-_12_final	7a	371382275	116571400	299236025	40.2	59.808	2.8	2980.63	690.13	1476.13	36.17	63.83	2.48	64.0314
chromosome_7_-_7_final	7b	373772025	137440875	236331150	36.8	63.229	2.5	2779.43	995.73	1783.7	35.82	64.18	2.31	64.4434
chromosome_8_-_46_final	8a	329942800	149268625	222113650	19.4	80.648	2.5	2369.78	1078.28	1902.35	32.18	67.82	1.97	56.8866
chromosome_8_-_10_final	8b	368566000	81523750	287042250	22.1	77.881	2.4	2580.2	840.25	1739.95	32.56	67.44	2.14	63.5458
chromosome_9_-_8_final	9a	269799750	107572025	238645275	33.8	66.232	2.3	2618.01	743	2360.99	32.73	67.27	2.18	46.5171
chromosome_9_-_24_final	9b	346217300	87104875	250252200	31.1	68.93	2.2	3103.99	1498.37	2201.15	23.93	76.07	2.58	59.6926
chromosome_10_-_1_final	10a	324328400	63851700	266091100	20.2	79.8	2.2	1999.6	762.74	1607.04	23.9	76.1	1.66	55.918
chromosome_10_-_13_final	10b	313586625	88393525	240548000	35.6	64.396	2.2	2341.92	1181.39	1450.83	38.39	61.61	1.95	54.0666
chromosome_11_-_25_final	11a	328941525	65500325	258828075	26.9	73.128	2.1	2632.22	477.95	1521.65	44.88	55.12	2.19	56.714
chromosome_11_-_36_final	11b	337357075	117128000	197302600	25.8	74.181	2.1	3699.52	1871.05	1310.87	40.5	59.5	3.08	58.165
chromosome_12_-27_final.	12a	255715350	111649725	201936900	23.4	76.634	2.1	2768.35	899.07	1442.85	33.27	66.73	2.3	44.0888
chromosome_12_-14__	12b	287741025	55935275	235704975	26.1	73.906	1.9	2627.77	515.58	1196.24	26.25	73.75	2.188	49.6105
chromosome_13_-_2_final	13a	213495425	75083525	212657500	11.8	88.151	1.9	2016.5	690.05	1937.72	13.34	86.66	1.67	36.8095
chromosome_13-_38_final	13b	238463775	91106950	178692800	9.6	90.415	1.8	2037.57	857.13	1760.88	16.49	83.51	1.697	41.1144
chromosome_14_-_3_final	14a	187441100	102520275	164950225	16.8	83.218	1.8	1910.41	878.57	1443.88	24	76	1.59	32.3174
chromosome_14_-_26_final	14b	241776150	59752825	195962525	14.1	85.874	1.7	1807.73	921.24	1847.11	11.63	88.37	1.5	41.6855
chromosome_15_-_31_final	15a	231563750	34155275	207620875	16.6	83.367	1.6	2120.93	210.26	1597.47	26.07	73.93	1.76	39.9247
chromosome_15_-_37_final	15b	188212475	22856900	215606875	19.7	80.309	1.6	1810.24	336.16	1701.41	27.9	72.1	1.5	32.4504
chromosome_16_-_44_final..	16a	267470500	75119825	161008650	38.3	61.671	1.6	2322.45	740.94	1294.86	37.82	62.18	1.93	46.1156
chromosome_16_-_41_final	16b	422643925	38517325	193046425	35.4	64.648	1.5	2166.26	553.08	1567.85	31.85	68.15	1.8	72.8696
chromosome_17_-_21_final	17a	181872075	25298075	188197350	34.9	65.105	1.4	1645.65	269.09	1747.41	36.06	63.94	1.37	31.3572
chromosome_17_-_39_final	17b	236128475	41896250	170489000	31.8	68.187	1.4	2035.8	599.28	1322.39	36.39	63.61	1.695	40.711
chromosome_18_-_33_final	18a	212385250	85556075	105139925	19.7	80.274	1.3	1921.67	742.05	796.86	31.18	68.82	1.6	36.6181
chromosome_18_-_45_final	18b	291640250	37062300	151150175	19.2	80.821	1.2	1711.82	505.2	1305.04	30.11	69.89	1.42	50.2828
chromosome_19_-_28_final	19a	112569325	31456975	155984125	47.9	52.09	1.2	1520.94	458.58	1451.83	48.69	51.3	1.26	19.4085
chromosome_19_-_6_final	19b	126871525	63464500	118407575	45.1	54.93	1.2	1733.76	593.43	1052.22	49.08	50.92	1.44	21.8744
chromosome_20_-_43_final	20a	114006200	57184600	69686925	37.9	62.102	0.8	1449.02	882.91	850.85	46.8	53.2	1.2	19.6562
chromosome_20_-_5_final	20b	190696000	21187100	104468375	44.9	55.135	0.8	1538.91	866.93	1891.44	48.21	51.79	1.28	32.8786
chromosome_21_-_19_final	21a	75440475	43206075	70800125	18.0	81.985	0.8	1475.33	678.15	770.87	24.13	75.87	1.22	13.0069
chromosome_21_-11_final	21b	86747925	53932725	58636600	23.7	76.257	0.7	1310.35	740.67	780.27	36.11	63.89	1.09	14.9565
chromosome_22_-_35_final	22a	105871975	31475125	74396850	29.7	70.271	0.7	1057.13	402.51	654.62	38.07	61.93	0.88	18.2537
chromosome_22_-_9_final	22b	92761625	26145075	66616550	28.2	71.815	0.6	1023.41	294.14	729.27	28.74	71.26	0.85	15.9933
chromosome_X_-__40_edited_new	(X) 23	314430600	20597225	66150700	37.3	62.75	0.6	3181.92	473.19	837.16	41.2	58.8	2.65	54.2121
chromosome_Y_-_32_final	(Y) 24	125655475	13591325	61849150	16.9	83.139	0.5	2758.37	356	1119.33	31.43	68.57	2.3	21.6647
**Total**		**15085142600**					**100.00**	**120046.57**					**99.83**	

Supplementary Table S1. Measurements of chromosomes after narrow segmentation. Summary table showing raw data for volume and length measurements for all 46 narrow segmented prophase chromosomes obtained from Avizo software. Calculations for p and q arm %, whole chromosome % and DNA content are given in Supplementary calculations (S1, S2 and S4).

Material number	Resolved Chromosome number	Chromosome volume (nm³)	Calculated DNA content (Mbp)	CenterX (nm)	CenterY (nm)	CenterZ (nm)	Radius/MEMB (nm)
chromosome_1_4_combined	1a	1340912925	231.19	10618.946	20902.945	6659.7163	3326
chromosome_1_42_combined	1b	1599589750	275.79	11644.166	20413.619	3772.0825	3345
chromosome_2_34_combined	2a	1344812150	231.86	8554.6309	19515.209	5801.5547	2062
chromosome_2_22_combined	2b	1325101250	228.46	7463.1465	18429.328	5816.7554	2481
chromosome_3_29_combined	3a	1082759425	186.68	11467.317	16246.604	6685.4697	2808
chromosome_3_17_combined	3b	1181114275	203.64	9664.0703	20248.027	4075.1323	2527
chromosome_4_20_combined	4a	1248547575	215.266	7519.7549	18491.967	2953.1899	3281
chromosome_4_16_combined	4b	1221113850	210.536	7321.1499	19342.553	3555.5393	3301
chromosome_5_30_combined	5a	873943675	150.67	12443.127	16150.644	6099.0591	3317
chromosome_5_18_combined	5b	970550075	167.33	9795.4863	18826.332	2738.593	2620
chromosome_6_15_combined	6a	974942375	168.09	9164.1514	17051.939	2408.9419	3049
chromosome_6_23_combined	6b	932507675	160.77	8391.7148	17831.779	4130.8047	1820
chromosome_7_12_combined	7a	904453825	155.94	12082.962	18069.578	4741.6875	2300
chromosome_7_7_combined	7b	920943100	158.78	9958.002	18296.07	8247.1055	3037
chromosome_8_46_combined	8a	828205675	142.794	11395.227	19114.521	7020.4619	2623
chromosome_8_10_combined	8b	950742375	163.92	11293.394	20623.805	5300.4062	2998
chromosome_9_8_combined	9a	843742075	145.47	8832.0059	16114.48	7730.083	3294
chromosome_9_24_combined	9b	861644025	148.55	8710.6016	20613.592	5841.3682	2906
chromosome_10_1_combined	10a	606618375	140.1	10229.476	19518.533	8080.7876	3255
chromosome_10_13_combined	10b	781436150	134.73	12664.235	19195.689	5547.1899	3086
chromosome_11-25_combined	11a	774663175	133.56	10188.726	17243.354	7023.5005	1984
chromosoem_11_36_combined	11b	798149275	137.61	12262.213	18628.455	7966.0947	3713
chromosome_12_27_combined	12a	692355950	119.37	8412.2676	18135.445	6597.7705	1981
chromosome_12_14_combined	12b	774971725	133.61	11506.471	17753.301	7505.7173	2838
chromosome_13_2_combined	13a	633652800	109.25	10273.442	17865.865	5117.3169	473
chromosome_13_38_combined	13b	605121000	104.33	9724.5742	18105.484	1737.3898	3492
chromosome_14_3_combined	14a	595259500	102.63	11124.825	17393.752	4060.9155	1843
chromosome_14-26_combined	14b	629841300	108.59	10619.313	19079.908	5962.1445	1520
chromosome_15_31_combined	15a	527647725	90.97	9963.1543	21162.957	5254.2305	3160
chromosome_15_37_combined	15b	549954075	94.819	11028.442	18494.551	2870.9792	2685
chromosome_16_44_combined	16a	664737700	114.6	12378.194	19470.723	4559.1138	3010
chromosome_16-41_combined	16b	852520625	146.98	11729.229	19118.195	3371.3601	2875
chromosome_17_21_combined	17a	552482975	95.255	9488.1924	19402.152	5563.043	1478
chromosome_17-39_combined	17b	595465200	102.66	8904.0029	19655.193	3719.1455	2421
chromosome_18_33_combined	18a	441713525	76.15	12222.978	20520.375	6738.0664	3783
chromosome_18_45_combined	18b	519204950	89.51	10353.921	20735.186	4009.9011	3033
chromosome_19_28_combined	19a	319603350	55.1	11001.119	15699.03	4639.0366	2650
chromosome_19_6_combined	19b	321245925	55.38	10594.163	19183.908	4354.3091	1651
chromosome_20_43_combined	20a	270513650	46.64	10382.965	19169.367	2806.9492	2740
chromosome_20_5_combined	20b	391834300	67.55	9341.8633	20428.834	7704.5405	3501
chromosome_21_19_combined	21a	247720275	42.71	8425.8857	18840.818	5090.5288	1644
chromosome_21_11_combined	21b	260431325	44.9	11248.175	18673.49	4636.7568	1670
chromosome_22_35_combined	22a	286379775	49.37	10381.077	18209.836	6159.3364	1100
chromosome_22_9_combined	22b	256807375	44.277	9317.1367	19341.57	4513.46	1601
chromosome_X_combined	(X) 23	728537975	125.6	9267.6523	17432.012	6748.3257	1723
chromosome_Y_combined	(Y) 24	304665900	52.52	10963.175	20246.205	7961.2949	3711
Membrane__final		2537239925		9835.2021	18005.523	5226.3589	891

Supplementary Table S2: Medium segmentation of chromosomes. Summary table showing raw data for volume and length measurements for all 46 wide segmented prophase chromosomes obtained from Avizo. Calculation for DNA content and distance from center of the nucleus are given in Supplementary calculations S1, S2, S4 and S6.

Material number	Resolved Chromosome number	Chromosome volume (nm³)	Calculated DNA content (Mbp)
chromosome_1_-_42_vws	1b	2724369450	469.7188707
chromosome_8_-_10_vws	8b	1288175075	222.0991509
chromosome_16_-_44_vws	16a	872688300	150.4635
chromosome_18_-_45_vws	18b	825241175	142.2829612

Supplementary Table S3: Wide segmentation of chromosomes. Summary table showing raw data for volume and DNA content measurements for 4 wide segmented prophase chromosomes obtained from Avizo. Calculation for DNA content of chromosomes is given in Supplementary calculations S4.
