# Supplementary material for: Ultra-Structural Imaging Provides 3D Organization of 46 Chromosomes of a Human Lymphocyte Prophase Nucleus
Source: Int J Mol Sci. 2021 Jun 1;22(11):5987. doi: 10.3390/ijms22115987 (PMC8198510; doi:10.3390/ijms22115987)
Supplement: Supplementary file 1 [file ijms-22-05987-s001.zip › Supplementary Calculations.pdf]

Supplementary Calculations

S1- Volume Measurement in 3D

p arm volume (centromere index) = Total volume of chromosome – q arm volume

% of p arm volume (centromere index) = p arm volume of individual chromosome / total volume of individual chromosome x 100

% of q arm volume = q arm volume of individual chromosome / total volume of individual chromosome x 100

% volume of whole individual chromosome = volume of individual chromosome / total volume of all chromosomes x 100

S2- Length Measurement in 3D

% of p arm length (centromere index) = p arm length of individual chromosome / total length of individual chromosome x 100

% of q arm length = q arm length of individual chromosome / total length of individual chromosome x 100

% length of whole individual chromosome = length of individual chromosome / total length of all chromosomes x 100

S3- Length Measurement in 2D (mFISH spreads)

% of p arm length = p arm length of individual chromosome / total length of individual chromosome x 100

% of q arm length = q arm length of individual chromosome / total length of individual chromosome x 100

Total averaged length of 5 spreads = sum of averaged lengths of 5 spreads / 5

% length of whole individual averaged chromosome of 5 spreads = Total Averaged length of individual chromosomes from 5 spreads / total lengths of all averaged chromosomes (5 spreads) x 100

S4- Calculation for DNA content of chromosomes

Number of base pair (bp) per chromosome = volume of chromosome (nm^3) / 5.80 (nm^3/ bp)

Number of Mega base pair (Mbp) per chromosome = number of bp per chromosome / 1000000

Where; 1 Mbp = 1000000 bp

S5- Diameter measurement of chromosomes

Diameter of whole chromosome p arm = Sum of p arm diameters of chromatid 1 and chromatid 2

Diameter of whole chromosome q arm = Sum of q arm diameters of chromatid 1 and chromatid 2

Diameter of whole chromosome = Sum of p and q arm diameters of chromosome

Average chromosome diameter = Sum of diameters of 4 whole chromosomes / 4

S6- Calculation for finding center of chromosome position

dx = x - <x>, called offset x
dy = y - <y>
dz = z - <z>
r = sqrt(dx^2 + dy^2 + dz^2)

where <x> is the average x, so is the x coordinate of the center point, etc.
