# Supplementary material for: Ultra-Structural Imaging Provides 3D Organization of 46 Chromosomes of a Human Lymphocyte Prophase Nucleus
Source: Int J Mol Sci. 2021 Jun 1;22(11):5987. doi: 10.3390/ijms22115987 (PMC8198510; doi:10.3390/ijms22115987)
Supplement: Supplementary file 1 [file ijms-22-05987-s001.zip › Supplementary Video - revised/Supplementary Video S1 - Legend revised.pdf]

Supplementary Video S1: 3D visualization of a single prophase nucleus. 46 chromosomes are seen as single entities with their chromatids displaying as parallel to one another and with no twists.
